# Supplementary material for: The Role of Drones in Out-of-Hospital Cardiac Arrest: A Scoping Review
Source: J Clin Med. 2022 Sep 28;11(19):5744. doi: 10.3390/jcm11195744 (PMC9572186; doi:10.3390/jcm11195744)
Supplement: Supplementary file 1 [file jcm-11-05744-s001.zip › SupplementaryMaterialS1_SearchStrategy.pdf]

Supplementary Material S1: Search Strategy

Database	Search Strategy
**Medline**	1. OHCA.tw. or exp Out-of-Hospital Cardiac Arrest/<br>2. (out of hospital or out-of-hospital).tw.<br>3. exp Heart Arrest/ or ((heart or cardiac or cardiopulmonary or circulat* or ventricular or sudden) adj3 (arrest or death or standstill or asystole*)).tw.<br>4. 2 and 3<br>5. 1 or 4<br>6. drone*.tw.<br>7. ((unmanned or automat* or remote or remote-control* or robot* or mechani* or technolog*) adj3 (((aerial or airborne or flying or piloted) adj3 (vehicle? or device? or craft? or system? or network?)) or aircraft? or quadcopters?)).tw.<br>8. (robot* or machine* or technolog*).tw. or exp Technology/ or exp Aircraft/<br>9. ((deliver* or dispatch* or access* or deploy*) adj3 (AED* or defibrillator* or BLS* or basic life support*)).tw.<br>10. (pre-hospital or prehospital or ambulance? or emergency medical service? or EMS or emergency response system? or rescue or life saving).tw. or exp Emergency Medical Services/ or Disaster Medicine/<br>11. 6 or 7<br>12. 9 or 10<br>13. 8 and 12<br>14. 11 or 13<br>15. 5 and 14
**EMBASE**	1. ‘ohca’:ab,ti OR ‘out-of-hospital cardiac arrest’/exp<br>2. ‘out of hospital’:ab,ti OR ‘out-of-hospital’:ab,ti<br>3. ‘heart arrest’/exp OR (((heart or cardiac OR cardiopulmonary OR circulat* OR ventricular OR sudden) NEAR/3 (arrest OR death OR standstill OR asystole*)):ab,ti)<br>4. #2 AND #3<br>5. #1 OR #4<br>6. ‘drone’:ab,ti OR ‘unmanned aerial vehicle’/exp<br>7. ((‘unmanned’ OR ‘automat*’ OR ‘remote’ OR ‘remote-control*’ OR ‘robot*’ OR ‘mechanic*’ OR ‘technolog*’) NEAR/3 (‘aircraft*’)):ab,ti<br>8. ((‘unmanned’ OR ‘automat*’ OR ‘remote’ OR ‘remote-control*’ OR ‘robot*’ OR ‘mechanic*’ OR ‘technolog*’) NEAR/3 (‘aerial’ or ‘airborne’ or ‘flying’ or ‘piloted’) NEAR/3 (‘vehicle?’ OR ‘device?’ OR ‘craft?’ OR ‘system?’ OR ‘network?’)):ab,ti<br>9. (‘robot*’ OR ‘machine’ OR ‘technolog*’):ab,ti OR ‘robot’/exp OR ‘robotics’/exp OR ‘aircraft’/exp OR ‘quadcopter’/exp<br>10. (‘deliver*’ OR ‘dispatch*’ OR ‘access*’ OR ‘deploy*’):ab,ti AND (‘aed*’ OR ‘defibrillat*’ OR ‘bls*’ OR ‘basic life support’):ab,ti<br>11. (‘prehospital’ OR ‘pre-hospital’ OR ‘emergency medical service*’ OR ‘EMS’ OR ‘emergency response system’ OR ‘rescue’ OR ‘life saving’):ab,ti OR ‘emergency health service’/exp OR ‘disaster medicine’/de<br>12. #6 OR #7 OR #8<br>13. #10 OR #11

	14. #9 AND #13<br>15. #12 OR #14<br>16. #5 AND #15
**COCHRANE**	1. OHCA:ab, ti<br>2. MeSH descriptor: [Out-of-Hospital Cardiac Arrest] explode all trees<br>3. (out-of-hospital or out-of-hospital):ab,ti<br>4. MeSH descriptor: [Heart Arrest] explode all trees<br>5. ((heart or cardiac or cardiopulmonary or circular* or ventricular or sudden) NEAR/3 (arrest or death or standstill or asystole*)):ab,ti<br>6. #3 and (#4 or #5)<br>7. (#1 and #2) or #6<br>8. drone:ab,ti<br>9. ((unmanned or automat* or remote or remote-control* or robot* or mechani* or technolog*) NEAR/3 ((aerial or airborne or flying or piloted) NEAR/3 (vehicle? Or device? Or craft? Or system? Or network?)):ab,ti<br>10. (robot* or machine* or technolog*):ab,ti<br>11. MeSH descriptor: [Technology] explode all trees<br>12. MeSH descriptor: [Aircraft] explode all trees<br>13. #10 OR #11 OR #12<br>14. ((deliver* or dispatch* or access* or deploy*) adj3 (AED* or defibrillator* or BLS* or basic life support*)):ab,ti<br>15. (pre-hospital or prehospital or ambulance? or emergency medical service? Or EMS or emergency response system? or rescue or life saving:ab,ti<br>16. MeSH descriptor: [Emergency Medical Services] explode all trees<br>17: MeSH descriptor: [Disaster Medicine] explode all trees<br>18. #14 OR #15 OR #16 OR #17<br>19. #8 OR #9<br>20. #13 AND #18<br>21. #19 OR #20<br>22. #7 AND #21
**PSYCINFO**	1. OHCA.tw.<br>2. (out of hospital or out-of-hospital).tw.<br>3. exp Heart Disorders/ or ((heart or cardiac or cardiopulmonary or circula* or ventricular or sudden) adj3 (arrest or death or standstill or asystole*)).tw.<br>4. 2 and 3<br>5. 1 or 4<br>6. drone*.tw.<br>7. ((unmanned or automat* or remote or remote-control* or robot* or mechani* or technolog*) adj3 (((aerial or airborne or flying or piloted) adj3 (vehicle? or device? or craft? or system? or network?)) or aircraft? Or quadcopter?)).tw.<br>8. (robot* or machine* or technolog*).tw. or exp Technology/ or exp Aircraft/<br>9. ((deliver* or dispatch* or access* or deploy*) adj3 (AED* or defibrillator* or BLS* or basic life support*)).tw.<br>10. (pre-hospital or prehospital or ambulance? or emergency medical service? or EMS or emergency response system? or rescue or life saving).tw. or exp Emergency Services/ or Disaster Medicine/

	11. 6 or 7<br>12. 9 or 10<br>13. 8 and 12<br>14. 11 or 13<br>15. 5 and 14
**SCOPUS**	1. ohca OR "out-of-hospital cardiac arrest" OR ("out of hospital" or "out-of-hospital" AND ((heart OR cardiac OR cardiopulmonary OR circulat* OR ventricular OR sudden) W/3 (arrest OR death OR standstill OR asystole*)))<br>- AND -<br>2. drone* OR ((unmanned or automat* or remote or remote-control* or robot* or mechani* or technolog*) W/3 (((aerial or airborne or flying or piloted) W/3 (vehicle or device or craft or system or network)) or aircraft or quadcopter)) OR ((deliver* or dispatch* or access* or deploy*) W/3 (AED* or defibrillator* or BLS* or “basic life support”*)) OR ((pre-hospital or prehospital or ambulance or “emergency medical service” or EMS or “emergency response system” or rescue or “life saving”) or “Emergency Medical Services” or “Disaster Medicine” AND ((robot* or machine* or technolog*) or Technology or Aircraft))
