# Supplementary material for: The Role of Drones in Out-of-Hospital Cardiac Arrest: A Scoping Review
Source: J Clin Med. 2022 Sep 28;11(19):5744. doi: 10.3390/jcm11195744 (PMC9572186; doi:10.3390/jcm11195744)
Supplement: Supplementary file 1 [file jcm-11-05744-s001.zip › SupplementaryMaterialS3_DroneModelsandSpecifications.pdf]

Supplementary Material S3: Drone models and Specifications

Author	Year	Drone Model	Specifications
Claesson	2016	Two different eight-rotor class 2 UAVs from HEIGHT TECH GmbH & Co. KG company (DE)	Maximum velocity: 70 km/h<br>Maximum range: 10 km
Pulver	2016	From previous literature (Huston 2014)	Maximum velocity: 100 km/h
Rachunok	2016	Estimated	-
Claesson	2017	8-rotor drone developed and certified by the Swedish Transportation Agency	Maximum velocity: 75 km/h
Pulver	2018	From previous literature (Communication 2014)	Maximum velocity: 100 km/h
Bogle	2019	Estimated	Maximum velocity: 64.4 km/h<br>Maximum range: 19.3 km
Boutilier	2019	Estimated	Vertical acceleration: 9.81 m/s^2<br>Horizontal acceleration: 19.6 m/s^2<br>Maximum velocity: 100 km/h
Sanfridsson	2019	Modified DJI Inspire 1	
Cheskes	2020	Sparrow X1000 drone (Town of Caledon)<br>InDro M210C drone (County of Renfrew)	Sparrow X1000<br>Maximum velocity: 80 km/h<br>Maximum range: 25 km<br>InDro M210C<br>Maximum velocity: 55 km/h<br>Maximum range: 25 km
Glick	2020	Multi-copter MD4-3000	Cruising speed: 72 km/h<br>Maximum range: 50 km
Lancaster	2020		Maximum velocity: 100 km/h
Mackle	2020	Drone developed by Delft University of Technology	Maximum velocity: 100 km/h<br>Maximum range: 12 km
Rosamond	2020	DJI Matrice 600 Pro hexacopter	-
Sedig	2020	Sparrow X1000 drone	Maximum velocity: 80 km/h<br>Maximum range: 25 km
Starks	2020	Unspecified	-
Starks	2020	-	-
Zegre Hemsey	2020	Unspecified	-
Bauer	2020	Estimated	Multirotor systems<br>Maximum velocity: 50 km/h<br>Tiltwing or tiltrotor systems<br>Maximum velocity: 100 km/h

Chu	2020	From previous literature (Boutilier 2019)	Vertical acceleration: 9.81 m/s^2<br>Horizontal acceleration: 19.6 m/s^2<br>Maximum velocity: 100 km/h
Derkenne	2020	Estimated	Cruising speed: 80 km/h
Ryan	2021	Eagle drone model by Flirtey	Maximum velocity: 48.3 km/h<br>Maximum range: 16.1 km
Schierbeck	2021	DJI Matrice 600 Pro hexacopter	Maximum velocity: 60 km/h<br>Maximum range: 6 km
Schierbeck	2021	DJI Matrice 600 Pro hexacopter	Maximum velocity: 60 km/h<br>Maximum range: 6 km
Choi	2021	Huesin Blueye 1 k model (Huins Inc.)	Maximum velocity: 50 km/h
Rees	2021	Penguin B fixed wing drone	Indicated velocity: 90 km/h<br>Maximum range: 80.5 km
Baumgarten	2021	Octocopter with operating system (type “Ceptor”, GlobeUAV)	Maximum velocity: 60 km/h
